# Novel Insights into the Transcriptome of *Dirofilaria immitis*

**DOI:** 10.1371/journal.pone.0041639

**Published:** 2012-07-23

**Authors:** Yan Fu, Jingchao Lan, Zhihe Zhang, Rong Hou, Xuhang Wu, Deying Yang, Runhui Zhang, Wanpeng Zheng, Huaming Nie, Yue Xie, Ning Yan, Zhi Yang, Chengdong Wang, Li Luo, Li Liu, Xiaobin Gu, Shuxian Wang, Xuerong Peng, Guangyou Yang

**Affiliations:** 1 Department of Parasitology, College of Veterinary Medicine, Sichuan Agricultural University, Ya’an, China; 2 The Sichuan Key Laboratory for Conservation Biology on Endangered Wildlife – Developing toward a State Key Laboratory for China, Chengdu Research Base of Giant Panda Breeding, Chengdu, Sichuan, China; 3 Department of Chemistry, College of Life and Basic Science, Sichuan Agricultural University, Ya’an, China; New England Biolabs, United States of America

## Abstract

**Background:**

The heartworm *Dirofilaria immitis* is the causal agent of cardiopulmonary dirofilariosis in dogs and cats, and also infects a wide range of wild mammals as well as humans. One bottleneck for the design of fundamentally new intervention and management strategies against *D. immitis* may be the currently limited knowledge of fundamental molecular aspects of *D. immitis*.

**Methodology/Principal Findings:**

A next-generation sequencing platform combining computational approaches was employed to assess a global view of the heartworm transcriptome. A total of 20,810 unigenes (mean length  = 1,270 bp) were assembled from 22.3 million clean reads. From these, 15,698 coding sequences (CDS) were inferred, and about 85% of the unigenes had orthologs/homologs in public databases. Comparative transcriptomic study uncovered 4,157 filarial-specific genes as well as 3,795 genes potentially involved in filarial-*Wolbachia* symbiosis. In addition, the potential intestine transcriptome of *D. immitis* (1,101 genes) was mined for the first time, which might help to discover ‘hidden antigens’.

**Conclusions/Significance:**

This study provides novel insights into the transcriptome of *D. immitis* and sheds light on its molecular processes and survival mechanisms. Furthermore, it provides a platform to discover new vaccine candidates and potential targets for new drugs against dirofilariosis.

## Introduction

The heartworm *Dirofilaria immitis* is the causal agent of cardiopulmonary dirofilariosis in dogs and cats, and also infects a wide range of wild mammals, as well as humans [1]–[6]. The incidence of dirofilariosis is increasing in temperate and tropical areas of the world [7]. Furthermore, on account of human and animal reservoir population dynamics and climate change, the accelerated introduction of new competent *Dirofilaria* vectors in non-endemic areas widens the distribution of this zoonosis [8], [9]. Adult worms reside in the pulmonary arteries and right ventricles, resulting in the production of blood-circulating microfilariae in dogs as natural hosts [7]. Although dogs with a low worm burden can be asymptomatic, higher burdens of adult heartworms can cause pulmonary arterial occlusion and inflammation, resulting in potentially fatal congestive heart failure [10]. Cases of human dirofilariosis are increasing, and hundreds of clinical cases have been reported to date. In some areas, the seroprevalence of *D. immitis* infections in humans can reach up to 30% [11]. Worms cannot reach maturity in humans, and immature worms are responsible for pulmonary, and in a few cases, encephalic, ocular, hepatic and testicular dirofilariosis in humans [7], [12]–[14].

For natural hosts, the current adulticidal therapies are not the first choice of treatment to reduce heartworm infection rates, due to the potential for severe thromboembolisms and perivascular inflammation [15]. Current effective control strategies are based on periodic chemoprophylaxis against microfilariae, L3 and L4 larvae to break the heartworm lifecycle [16], [17]. However, these are long-term therapies and not suitable for large infected populations. While avermectin-class drugs are widely used for prevention, the American Heartworm Society estimated that 27 million dogs remain untreated in the United States alone [18]. Besides, the use of individual and specific antigens for vaccination against dirofilariosis has not progressed substantially [19]. Therefore, both new preventive strategies such as vaccines and safer, more efficient curative adulticidal drugs are required.

One bottleneck for the design of radical new intervention and management strategies against *D. immitis* in the last decade was the limited knowledge of fundamental molecular aspects of *D. immitis* using advanced ‘-omic’ technologies, such as transcriptomics [20]. The initial survey of the adult heartworm transcriptome was achieved using traditional cloning and sequencing methods, and by generating and analyzing 4,005 expressed sequence tags (ESTs) [21]. However, after trimming and assembly, only 1,793 EST clusters remained. Next-generation sequencing technology, such as Solexa/Illumina, Roche 454 and SOLiD platforms, has dramatically improved the efficiency of gene discovery [22]. It has produced a high coverage of expressed sequences within contigs [23], and also made the detection of low abundant transcripts possible [24]. To date, several nematode transcriptomes have been sequenced by using the next-generation sequencing technology, including *Trichuris suis*, *Necator americanus*, *Brugia malayi*, *Trichostrongylus colubriformis*, etc [25]–[30].

In the absence of comparative analyses of assembled EST datasets across nematode species, research on single species is limited in its practical use in identifying species-specific or species group-specific genes and associating these with features of the species biology or pathogenesis for further investigations of novel targets for antihelminthic drugs and potential vaccines. Furthermore, comparisons among species-groups can filter evolutionary conserved patterns from the background of neutral variation to identify biochemical or regulatory pathways uniformly absent or present in groups of species [11], [31], [32]. These analyses can contribute to interpret potential host-parasite interactions, the evolution of parasitism, as well as core biology and particular adaptations specific to parasitism (reviewed in [31], [33], [34]).

In the present study, a next-generation sequencing platform and powerful *de novo* short-read assembly was employed to uncover a global view of the heartworm transcriptome, which produced over 10 times more unique genes than obtained by previous studies [21]. The data were subjected to detailed bioinformatic analyses. Combining the intestinal-expressed transcriptomes from three nematode species [35], as well as the EST-derived transcriptomes data of over 60 species of nematodes from NEMBASE4 [31], we conducted for the first time a comprehensive comparative transcriptomic study to mine potential intestinal-expressed *D. immitis* molecules, and filarial-specific genes as well as genes with function potentially involved in filarial-*Wolbachia* symbiosis.

## Methods

### Ethics Statement

The animal from which specimens were collected, was handled in accordance with animal protection law of the People’s Republic of China (a draft of an animal protection law in China released on September 18, 2009). The owner of the dead dog gave permission to use tissue. This study was approved by the National Institute of Animal Health Animal Care and Use Committee at Sichuan Agricultural University (approval number 2010–020).

### Parasite Material

Live adult heartworms were collected by necropsy of an adult dog with sudden death, obtained from a veterinary hospital in Ya’an, Sichuan, China. Phosphate-buffered saline (PBS; pH 7.4; 37°C) was used five times to clean the live worms, both males and females, to remove host contamination. Then the worms were immediately frozen in liquid nitrogen and stored at −80°C until further use. No special measures were undertaken to remove developing embryos or sperm from the worms; therefore it was expected that the spermatic and embryonic transcripts existed in the present transcriptome.

### cDNA Library Construction and Illumina Sequencing

Total RNA was extracted with TRIzol (Invitrogen) from four whole worms of both sexes (two males and two females), which were ground in liquid nitrogen. The integrity of total RNA was verified using Agilent 2100 with an RNA integrity number (RIN: 8.0). An OligoTex mRNA mini kit (Qiagen) was used to isolate poly (A) mRNA after total RNA was collected from the heartworms, according to the manufacturer’s protocol. Divalent cations were used to fragment the purified mRNA into small pieces at 94°C for 5 minutes, thereby avoiding priming bias when synthesizing cDNA. The cleaved RNA fragments were used for double-stranded cDNA synthesis using a SuperScript Double-Stranded cDNA Synthesis kit (Invitrogen, Camarillo, CA, USA) with random hexamers (N6) primers (Illumina). The synthesized cDNA was subjected to end repair process and ligation of adaptors. These products were purified using a 2% TAE-agarose gel (Certified LowRange Ultra Agarose, Bio-rad) and enriched with PCR to create the final cDNA library with a size of approximately 200 bp. After detection by an Agilent 2100 Bioanalyzer, the cDNA library was sequenced from both 5′ and 3′ ends on a PE flow cell using HiSeq™ 2000 (Illumina), by Beijing Genomics Institute (BGI)-Shenzhen, Shenzhen, China, according to the manufacturer’ s instructions (Illumina, San Diego, CA, USA).

### De novo Assembly

Prior to assembly, a stringent filtering process was carried out to remove adaptor sequences, reads containing more than 5% ‘N’ rate (the ‘N’ character representing ambiguous bases in reads), low quality reads with more than 10% Q-value <20, and reads composed of more than 33% adenine, which were suspected of containing poly-adenine tails. Because we perform the mRNA purification by poly-A selection, it is presumably that no contamination from *Wolbachia* appeared in the data due to the lack of poly A tails in bacterial transcripts. To test our hypothesis, we queried the clean reads against the genome of *Wolbachia* endosymbiont strain TRS of *Brugia malayi* (NCBI Reference Sequence: NC_006833). *de novo* assembly of the clean reads was performed by using the Trinity program, which was designed specifically for transcriptome assembly [36]. Briefly, Trinity first combines reads of a certain length of overlap to form longer fragments without N (gaps), which are called contigs. These contigs will be further processed for sequence clusters with the sequence clustering software TGICL [37], and these sequences are defined as unigenes. The calculation of unigene expression used the RPKM method [38], which was able to eliminate the influence of different gene lengths and sequencing discrepancy on the calculation of gene expression.

### Host-associated Transcripts Contamination Analyses

To test whether our heartworm transcriptome data were polluted by the host-associated transcripts, we firstly queried our unigenes against 22,131 canine-derived protein-coding sequences from NCBI Genome dataset (Accession No. PRJNA12384) by BLAST, using an e-value of 1.0 e-5 as a cut-off, to identify putative homologs. Secondly, we queried these unigenes against all the EST sequences of nematodes in NCBI (e-value of 1.0 e-5). The unigenes which can only matched to canine-derived sequences but nematode-derived sequences might come from the host.

### Functional Assignment

To obtain as many functional annotations for *D. immitis* transcriptome as possible, we annotated the assembled unigenes (longer than 300 bp) based on two levels of sequence similarity, namely sequence-based and domain-based alignments, as previously described [24]. For assignments of predicted gene descriptions, unigene sequences first were aligned by BLASTx to the animal protein datasets of NR, UniProtKB/Swiss-Prot, UniProtKB/TrEMBL, and NEMBASE4 (a nematode transcriptome database) (e-value <0.00001), retrieving proteins with the highest sequence similarity with the given unigenes along with their protein functional annotations. The pathway annotation was carried out according to the Kyoto Encyclopedia of Genes and Genomes (KEGG) pathway database [39], also using BLASTx with an e-value threshold of 10^−5^. InterPro database (version 33.0) [40], Pfam database (version 25.0) [41] and the clusters of orthologous groups (COGs) database at NCBI [42] were employed in domain-based comparisons with the assembled unigenes (e-value <0.00001), as previously described [24]. In addition, the best aligning results from the above protein databases were used to produce the putative gene names, ‘CDS’, as well as to decide the orientation of unigenes. When unigenes happened not to be aligned to any of the above databases, ESTScan program (version 3.0.1) was used to predict the ‘CDS’ and sequence direction of them.

Based on the best BLASTx hits from the animal protein datasets of the NR database, the Blast2GO program [43] was used to obtain GO annotations (e-value <0.00001) according to molecular, function, biological process, and cellular component ontologies. Afterwards, WEGO software [44] was employed to perform GO functional classification for all unigenes and to interpret the distribution of gene functions of heartworms on a macroscopic level.

### Identification of Intestinal Transcriptome

To mine potential intestine-associated genes, we downloaded the datasets of intestine-expressed genes from three nematode species, respectively: A) 9,586 ESTs from *Ascaris suum* intestinal libraries in Genbank; B) 7,078 ESTs from *Haemonchus contortus* intestinal libraries in Genbank, according to [35]; C) a set of 5,438 *Caenorhabditis elegans* intestinally-expressed genes were consolidated from two previous studies: C1) over 4,000 *C. elegans* intestinal genes discovered by mapping with SAGE (serial analysis of gene expression) data from the dissected adult intestine [45], [46], and C2) about 1,900 intestine-expressed genes obtained by using mRNA tagging and microarray gene expression profiling [47]. The 9,586 *A. suum* ESTs and 7,078 *H. contortus* ESTs were then assigned into 3,042 and 1,659 EST clusters, respectively, as described before [21]. We then queried our unigenes against the intestinal-expressed genes from these datasets by BLASTn, using an e-value of 1.0 e^−5^ as a cut-off, to identify putative homologs in these nematodes.

### Comparative Sequence Analysis within NEMBASE4

NEMBASE4 presents a single portal into extensively functionally annotated, EST-derived transcriptomes of 62 nematode species, with nearly 700,000 ESTs and 240,000 putative transcripts which have been filtered both for length (>100 bases) and quality [31]. Datasets used for sequence comparisons were four groups of EST-clusters downloaded from the NEMBASE4 dataset [31]. The nematode species included in each group are shown in Table S1. BLASTn was used to query our unigenes against these datasets. An e-value of 1.0 e^−5^ and identity ≥80% was used to accept sequence similarities.

### Accession Numbers

The transcriptome datasets (raw reads) are available at the NCBI Short Read Archive under the accession no. SRA048975. The assembled unigenes (longer than 300 bp) are available from Transcriptome Shotgun Assembly Sequence Database (TSA) at NCBI with the following accession numbers: JR895929–JR916738.

## Results

### Illumina high-throughput Sequencing and de novo Assembly

As shown in Table 1, a total of 22,609,857 raw reads of 90 bp length were obtained by HiSeq™ 2000 (Illumina) paired-end sequencing. After a stringent filtering process, 22,250,630 high-quality clean reads remained. The Q20 percentage (sequencing error rate <1%), and GC percentage were 92.72% and 38.17%, respectively. The analysis of *Wolbachia*-contamination showed that 0.04% clean reads were mapped to *Wolbachia* genome, and only 12 reads were mapped to *Wolbachia* genes (summarized in Table S2). Based on the clean reads, a total of 25,824 contigs (≥300 bp) without gaps was produced by Trinity, a short reads assembling program. The average length and N50 of these contigs were 1,388 bp and 2,021 bp, respectively. After clustering using TGICL software, the 25,824 contigs generated 20,810 distinct sequences (containing no gaps) with 2,978 clusters (mean length of 2,021 bp) and 17,832 singletons (mean length of 1,145 bp). These distinct sequences were unigenes, ranging from 300 bp to 16,275 bp, with a mean length of 1,270 bp and N50 of 1,852 bp. Of the 20,810 unigenes, 1,489 unigenes (71.6%) were ≥500 bp and 9,401 unigenes (45.2%) were ≥1,000 bp. Assessment of assembly is necessary in transcriptomic analysis. The quality of our assembly had been critically assessed by Beijing Genomics Institute, (BGI)-Shenzhen, before the subsequent analysis. Several analytic approaches described by Wheat and Vogel [48], such as the Sequencing depth (base number on mapped reads/unigene sequence length); the Coverage (base number on unigene covered by reads/unigene sequence length); Unique-mapped-Reads (the Number of unique mapped reads per assembled unigene), were taken into our quality assessment. The detail information about assessment is shown in Table S3.

**Table 1:** Summary of transcriptome data for adult *Dirofilaria immitis* prior to and following assembly as well as detail bioinformatics annotation and analyses.

Raw reads (paired-end)	22,609,857
clean reads (paired-end)	22,250,630
GC content percentage	38.17%
Contigs (≥300 bp) (average length; N50 )	25,824 (1,388; 2,021)
Singletons (≥300 bp) (average length; N50 )	17,832 (1,145; 1,674)
Clusters (≥300 bp) (average length; N50 )	2,978 (2,021; 2,582)
Total unigenes (average length; N50; min-max length)	20,810 (1,270; 1,852; 300–16,275)
mean RPKM value of unigenes (min-mix RPKM value)	32,93 (0–6,617.22)
Protein coding sequence (CDS)	15,698
Gene annotation against Animals protein of Nr (%)	15,602 (75.0)
Gene annotation against UniProtKB/Swiss-Prot (%)	11,481 (55.2)
Gene annotation against UniProtKB/TrEMBL (%)	15,659 (75.2)
Gene annotation against Nemabse4 (%)	14,093 (67.7)
Gene annotation against KEGG (%)	9,139 (43.9); 3,704 KO terms; 216 biological pathways
Gene annotation against InterPro (%)	16,729 (80.3); 4,229 domains/families
Gene annotation against Pfam (%)	10,839 (52.1); 3,247 domains/families
Gene annotation against COG (%)	5,604 (26.9); 1,322 COG functional terms
All annotated unigenes (%)	17,719 (85.1)
GO Ontology (%)	2,930 (14.1); 1,637 GO terms
Biological process category	2,334; 1,045 GO terms
Cellular component category	1,754; 373 GO terms
Molecular function category	1,940; 219 GO terms

### Host-associated Transcripts in D. Immitis

Analyses of host-associated transcripts contamination revealed that 617 unigenes were shown to contain similarities (e-value <0.00001 and identity ≥80%) with the canine genes (data not shown). Among them, only 34 unigenes (0.16% of total unigenes) failed to have BLAST matches (e-value <0.00001 and identity ≥80%) with genes derived from nematodes in NCBI (File S1), which indicated that there was little gene contamination from the host in our transcriptome data. Further studies are needed to confirm the host origin. However, we did not filter the 34 unigenes from our dataset, because they also potentially represent homologous genes only shared between heartworms and their host.

### Functional Annotation and Classification by COG

**Figure 1:**
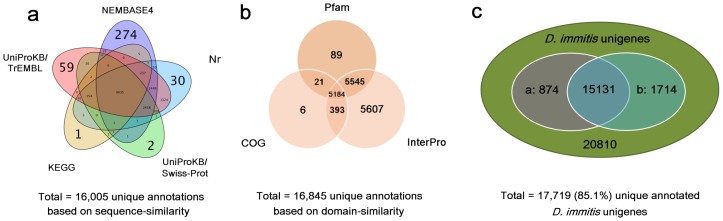
Venn diagram illustrating distribution of high-score matches among eight public databases. (a) By integrating sequence-similarity search results from the animal protein dataset of the Nr, UniProtKB/Swiss-Prot, UniProtKB/TrEMBL, KEGG, and NEMBASE4 databases, a total of 16,005 unigenes were returned with unique best BLASTx hits (e-value <0.00001). (b) By integrating sequence-similarity search results from InterPro, Pfam and COG databases, a total of 16,845 unigenes obtained unique domain-based annotations (e-value <0.00001). (c) Consolidating from both unique sequence-based annotations and unique domain-based annotations produces 17,719 unique annotated unigenes. The ellipses ‘a’ and ‘b’ imply the two subsets of *D. immitis* unigenes (16,005 counts in Figure 1a; 16,845 counts in Figure 1b).

We obtained 15,602 (75.0%), 11,481 (55.2%), 15,659 (75.2%), 14,093 (67.7%), 9,139 (43.9%) high-score BLAST matches against NR database, UniProtKB/Swiss-Prot, UniProtKB/TrEMBL, NEMBASE4 and KEGG, respectively (Table 1; Fig. 1a). In total, there were 8,635 (41.5%) unigenes showing hits in all five databases with relatively well-defined functional annotations. The results indicated that the longer unigenes were more likely to have BLAST matches in these databases. For instance, in the Nr database, all unigenes over 2,000 bp had significant BLAST matches and 98.8% for query unigenes between 1,500 and 2,000 bp, whereas the match efficiency decreased to 96.4% for those ranging from 1,000 to 1,500 bp, and to 75.4% for those between 500 and 1,000 bp (Fig. S1). Only 37.4% of the unigenes shorter than 500 bp could achieve significant BLAST scores.

**Figure 2:**
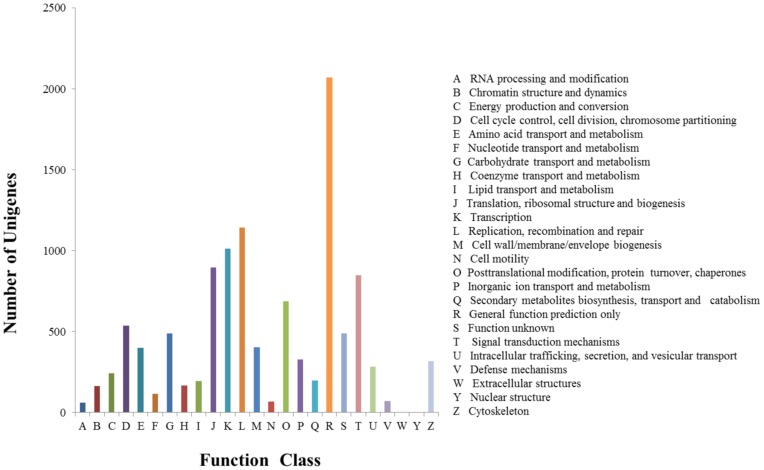
Histogram presenting clusters of orthologous groups (COG) classification. Of 20,810 unigenes, 5,604 sequences were assigned to 25 COG classifications.

Because certain protein domains can be shared by different genes, a level of sequence similarity exists not accounted for by simple sequence-based alignments [24]; therefore, protein domains inferred from the *D. immitis* transcriptome were further identified according to the presence of conserved (InterPro, Pfam and COGs databases) domains/signatures. In total, 16,729 (80.3%) unigenes matched entries in the InterPro database, and were categorized into 4,229 domains (Table 1; Fig. 1b). The 30 most commonly represented InterPro domains are shown in Table 2. Of these, the domains ‘Protein kinase, catalytic domain’, ‘Serine/threonine-protein kinase-like domain’ and ‘Protein kinase, ATP binding site’ were the highest ranked. These observations about the *D. immitis* kinase support the assumption that these molecules could present a unique opportunity for the design of pathogen-selective inhibitors through pharmacotherapy [14], [20], [24]. Similar analytical results were obtained by using the Pfam database. A total of 10,839 (52.1%) top hits were categorized into 3,247 domains by searching against the Pfam database (Table 1; Fig. 1b). The top five Pfam domains were ‘Protein kinase’, ‘RNA recognition motif’, ‘Tyrosine kinase’, ‘Nuclear receptor’ and ‘Zinc finger’ (Table S4). In addition, all unigenes were aligned in the COG database to predict and classify possible functions. In total, 5,604 (26.9%) sequences were assigned to 1,322 different COG functional terms (Table 1; Fig. 1b). A total of 19,895 COG functional annotations were produced, as some unigenes were annotated with multiple COG functions. As shown in Fig. 2, among the 25 COG categories, the cluster for ‘general function prediction’ represented the largest group (2,071, 18.5%), followed by ‘Replication, recombination and repair’ (1,142, 10.2%), ‘Transcription’ (1,012, 9%), ‘Translation, ribosomal structure and biogenesis’ (896, 8%), and ‘Signal transduction mechanisms’ (848, 7.6%). In total, 16,845 (80.9%) unigenes could be mapped to known proteins from the InterPro, Pfam, and COG databases (Fig. 1b).

**Table 2:** The 30 most represented (InterPro) protein domains/families in adult *Dirofilaria immitis* unigenes.

InterPro description	InterPro code	No. of unigenes
Protein kinase, catalytic domain	IPR000719	466
Serine/threonine-protein kinase-like domain	IPR017442	303
Protein kinase, ATP binding site	IPR017441	283
Tyrosine-protein kinase, catalytic domain	IPR020635	235
WD40/YVTN repeat-like-containing domain	IPR015943	224
Nucleotide-binding, alpha-beta plait	IPR012677	210
Zinc finger, RING/FYVE/PHD-type	IPR013083	196
Zinc finger, C2H2-type	IPR007087	191
WD40 repeat	IPR001680	190
Immunoglobulin-like fold	IPR013783	185
WD40 repeat, subgroup	IPR019781	175
RNA recognition motif domain	IPR000504	172
Zinc finger, C2H2-like	IPR015880	171
WD40-repeat-containing domain	IPR017986	134
Armadillo-like helical	IPR011989	128
Zinc finger, C2H2-type/integrase, DNA-binding	IPR013087	127
Serine/threonine-protein kinase domain	IPR002290	109
ATPase, AAA+ type, core	IPR003593	105
Tetratricopeptide-like helical	IPR011990	105
Nuclear hormone receptor, ligand-binding	IPR008946	101
Helicase, superfamily 1/2, ATP-binding domain	IPR014021	98
Zinc finger, RING-type	IPR001841	97
Pleckstrin homology-type	IPR011993	91
GPCR, rhodopsin-like superfamily	IPR017452	91
Nuclear hormone receptor, ligand-binding, core	IPR000536	90
Ankyrin repeat-containing domain	IPR020683	90
Ankyrin repeat	IPR002110	89
NAD(P)-binding domain	IPR016040	89
Zinc finger, nuclear hormone receptor-type	IPR001628	87
EF-hand-like domain	IPR011992	87

In conclusion, by integrating sequence-similarity search results from the Nr, UniProtKB/Swiss-Prot, UniProtKB/TrEMBL, KEGG and NEMBASE4 databases, a total of 16,005 unigenes (76.9% of all unigenes) were returned with unique best BLASTx hits. A Venn diagram (Fig. 1c) shows that an additional 1,714 unigenes were annotated by domain-based alignments. Altogether, 17,719 (85.1%) unigenes produced sequence-based or domain-based annotations by using the eight public databases (Table 1).

In addition, 15,560 protein-coding sequences (CDS) were inferred from these annotated unigenes. Another 138 CDSs were inferred from the remaining unigenes that could not be aligned to any above databases by using ESTscan. In total, 15,698 CDSs were obtained by consolidating the two efforts (Table 1), and the CDS length distribution is shown in Figs. S2a and S2b.

### Functional Classification by Gene Ontology (GO)

**Figure 3:**
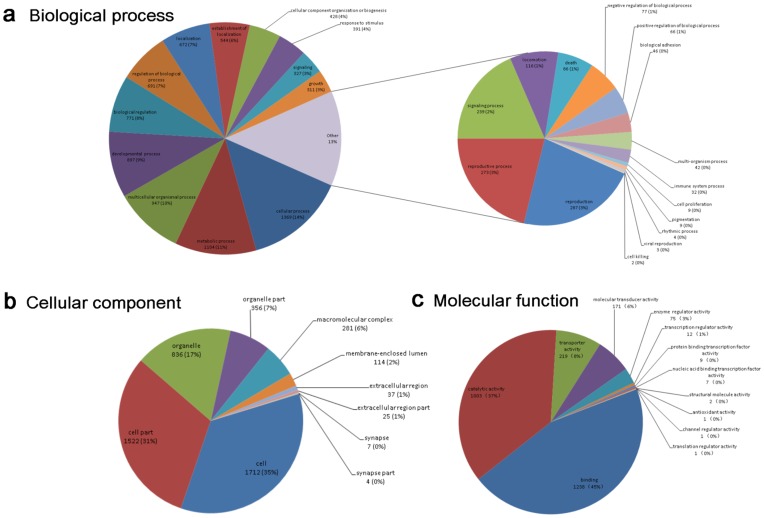
Pie charts showing gene ontology (GO) classification. The distribution of *D. immitis* unigenes in three main categories (‘biological process’, ‘cellular component’ and ‘molecular function’) are shown in the subgraphs a–b, respectively.

To give an overview of all the different functional classes in the heartworm transcriptome database, we were able to assign 1,637 different GO terms to 2,930 predicted peptides of *D. immitis* that were BLASTx-matched with known proteins in the Nr database. Specifically, 2,334 predicted proteins could be assigned to 1,045 ‘biological process’ terms, 1,754 to ‘cellular component’ terms and 1,940 to 219 ‘molecular function’ terms (Table 1). The predominant terms were ‘cellular process’ (GO: 0009987; 14%) and ‘metabolic process’ (GO: 0008152; 11%) for ‘biological process’; ‘cell’ (GO: 0005623; 35%) and ‘cell part’ (GO: 0044464; 31%) for ‘cellular component’; ‘binding’ (GO: 0005488; 45%) and ‘catalytic activity’ (GO: 0003824; 37%) for ‘molecular function’ (Fig. 3). Because we used adult worms as materials, with males full of sperm and females full of embryos, it is expected that a great part of transcripts in adult worms was reproductive in origin. Surprisingly, in the category ‘biological process’, only 273 (3%) and 287 (3%) predicted peptides were assigned to the classes named ‘reproductive process; GO:0022414’ and ‘reproduction; GO:0000003’, respectively. These unigenes involved in embryogenesis, spermatogenesis and egg production would be of significance in learning the reproductive biology of heartworms.

### Functional Classification by KEGG

To investigate the biological functions and interactions of genes, a pathway-based analysis was conducted using the KEGG Pathway database. As described above, 9,139 (43.9%) unigenes were assigned to 3,704 KEGG Orthology (KO) terms, and were further classified into six categories (‘Metabolism’, ‘Genetic information processing’, ‘Environmental information processing’, ‘Cellular processes’, ‘Organismal systems’, and ‘Human disease’) that comprised 216 KEGG pathways (Table 1; Table S5a, b). Surprisingly, the ‘Immune system’ sub-category, including 14 pathways, was associated with 600 (600/9,139; 6.6%) KEGG-annotated unigenes (Table S5a), which means that a much higher proportion of putative KO proteins of *D. immitis* is involved in pathways associated with the immune system than those of *Trichuris suis* (120/4,588; 2.6%) [29]. The diversity of quantity and types of those proteins referred from two species may reflect their different living conditions. Interestingly, a range of putative proteins of *D. immitis* were inferred to be involved in KEGG pathways of cardiovascular diseases (Table S6), including ‘Hypertrophic cardiomyopathy (HCM)’, ko05410 (n = 214); ‘Arrhythmogenic right ventricular cardiomyopathy (ARVC)’, ko05412 (n = 58); ‘Dilated cardiomyopathy’, ko05414 (n = 217); and ‘Viral myocarditis’, ko05416 (n = 156). By carefully checking the 34 ‘putatively contaminated’ unigenes, we found that none of them were relevant to the gene group associated with the human cardiomyopathy pathways. Heartworms release vasoactive substances that result in vasoconstriction and hypoxia, which could further lead to ventricular and endocardial hypertrophy, right ventricular dilatation, congestive heart failure or right heart failure [49]–[51]. These symptoms resulting from heartworm disease are, to some extent, similar to those of cardiovascular diseases, although their pathological mechanisms are significantly diverse. Given the host environment of heartworms, those unigenes may imply putative parasitism-related genes of heartworms that are adapted to the host’s heart. Further experimental validation and investigation is necessary to discover whether these heartworm unigenes are associated with the heart pathogenicity of hosts.

### Putative Intestinal-expressed Genes

**Figure 4:**
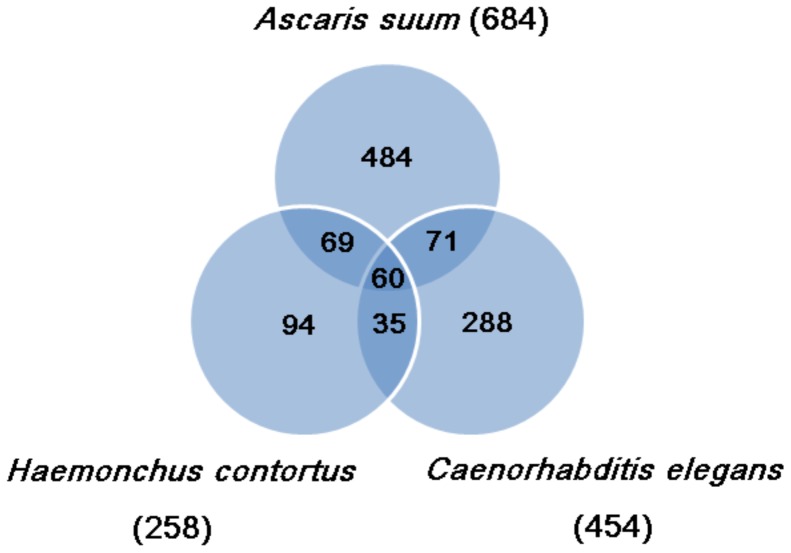
Distribution of sequence homology identified between heartworm unigenes and intestinal-expressed genes from three nematode species. A total of 1,101 heartworm unigenes (5.3% of the total yield) contained similarities (e-value <0.00001) identified by BLAST searches.

‘Hidden antigens’ derived from the intestinal epithelium of hematophagous parasites like heartworms, which are normally invisible to the hosts’ immune systems, but that are accessible to host immune response, would make better vaccine candidates than traditional exposed antigens like surface antigens and ES antigens [52], [53]. Therefore, the nematode intestine forms a key surface at the intestinal apical membrane that interacts with the environment. Furthermore, the adaptations at the apical intestinal membrane may play an important role in protecting parasitic nematodes against the host immune system; therefore, the intestine could be an important target for the control of nematodes [35]. To identify the intestinal transcriptome of *D. immitis*, we compared our unigenes to the intestinal transcriptomes from *A. suum* (3,042 ESTs), *H. contortus* (1,659 ESTs), and *C. elegans* (5,438 ESTs), respectively. As shown in Fig. 4, using an e-value of 1.0 e^−5^ as cut-off, 684,258,454 of our unigenes had high-score BLAST matches against intestinal genes from the three databases, respectively. Consolidating the three data sets provided us with a non-redundant set of 1,101 (5.3% of total *D. immitis* unigenes) putative intestinal genes from *D. immitis* (Table S7). Of the 1,101 unigenes in which similarities were detected, 60 contained matches to all three queried databases, which may include core intestinal functions that are indispensable for the survival of *D. immitis*. As shown in Fig. S2, 300 of the 1,101 unigenes could be assigned to 566 GO terms and further classified into 41 GO sub-categories by using WEGO. Additionally, 977 of the 1,101 unigenes could obtain KO annotations and were assigned to 175 KEGG biological pathways (Table S8). These annotations provide a valuable resource for further investigations of the specific functions of intestinal-expressed genes, as well as their role in heartworm-host interactions.

### Comprehensive Comparative Analysis Based on NEMBASE4

**Figure 5:**
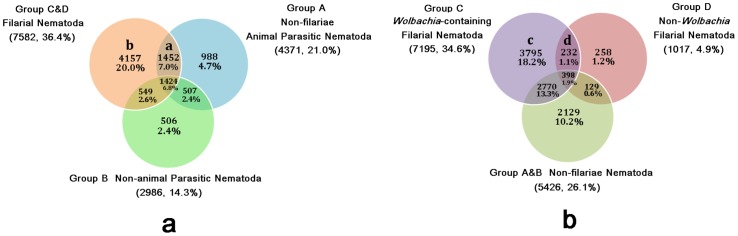
Distribution of sequence homology identified between heartworm unigenes and several groups of EST-clusters from NEMBASE4. (a) Group A includes 22 animal-parasitic nematode species without filarial species; Group B includes 29 other nematode species without animal-parasitic species (including eight free-living species, 18 plant-parasitic species and three entomopathogenic species); Group C&D includes 8 filarial species excluding *D. immitis*. (b) Group C includes six *Wolbachia*-containing filarial species excluding *D. immitis*; Group D includes two non-*Wolbachia* filarial species; Group A&B includes 51 non-filarial nematode species.

The present transcriptome of *D. immitis* could assist significantly in identifying genes linked specifically to parasitism and also to our understanding of the evolution of filarial species by combining cross-species comparative analysis. To further identify genes putatively unique to *D. immitis* or further to filarial species (Filarioidea), we queried our *D. immitis* unigenes against four groups of EST clusters from different species within NEMBASE4 (Table S1): (I) Group A: animal parasitic nematodes without filarial species, including 21 species; (II) Group B: Non-animal-parasitic nematodes (free-living nematodes, plant parasitic nematodes and entomopathogenic nematodes), including 29 species; (III) Group C: *Wolbachia*-containing filarial nematodes excluding *D. immitis*, containing six species; (IV) Group D: Non-*Wolbachia* filarial nematodes, including two species. In total, using an e-value of 1.0 e^−5^ and identity ≥80% as a cut-off, 13,676 (65.7%) *D. immitis* unigenes were shown to contain similarities to the EST clusters from other nematodes within NEMBASE4. Among them, 4,371 (21%); 2,986 (14.3%); 7,195 (34.6%); and 1,017 (4.9%) unigenes had significant matches with EST clusters from species of the four groups respectively. As shown in Fig. 5a, of the 13,676 (65.7%) unigenes in which similarities were detected, 1,424 (6.8%) simultaneously contained matches to EST sequences from other filarial nematodes (groups C & D), non-filarial animal parasitic nematodes (group A) and all non-animal parasitic nematodes (group B) simultaneously. This implied that these are highly conserved genes involved in basic physical activity in nematodes. In addition, 1,452 (7%) matches uniquely shared by group C & D and group A, represent potentially specific genes for animal parasitic nematodes, and were assembled into subgroup a (Table S9). Furthermore, 4,157 (20.0%) of matches contained similarities only to filarial nematodes (group C & D), which may contain putative filaria-specific genes. These 4,157 unigenes were gathered into subgroup b (Table S9).

Similar comparative analyses were also conducted to identify the overlap in identical matches between or among EST clusters from *Wolbachia*-containing filarial species (group C), non-*Wolbachia* filarial species (group D) and non-filarial nematodes (group A & B). As shown in Fig. 5b, there were 3,795 (18.2%) *D. immitis* unigenes (subgroup c) only matching to EST clusters from *Wolbachia*-containing filarial species, which may contain putative symbiosis-related genes adapted in these species or reflect the phylogenetic distance between *Wolbachia*-containing and non-*Wolbachia* filarial nematodes (Table S9). The well-studied genomes of some species, such as *Caenorhabditis* spp., can produce more annotated EST clusters than some species similar to *D. immitis*; therefore, the distribution of the matches most likely do not reflect the phylogenetic relationships among nematodes. Furthermore, 232 (1.1%) unigenes had significant matches with both groups C and D. The lack of similarities to EST clusters from extensively functionally annotated transcriptomes from all non-filarial nematodes suggests that such *D. immitis* unigenes represent specific molecules associated with parasitism or survival of filarial species. These 232 unigenes were assembled into subgroup d (Table S9). Surprisingly, 2,129 (10.2%) unigenes (data not shown) uniquely contained similarities to non-filarial nematodes, which may be due to incomplete genomes and the lack of representation of many filarial species.

To functionally categorize the unigenes within the four subgroups, corresponding GO annotations were used for GO classification. As a result, 284,630,578 and 33 unigenes from subgroups a–d, obtained 1,700, 3,346, 3,132 and 151 GO annotations, respectively, which were further assigned into 42, 41, 40, and 26 subcategories, respectively (Fig. S3a–d). To further analyze the biochemical or regulatory pathways associated with unigenes from subgroups a–d, respectively, we gathered the information about KEGG annotations and pathways derived from these unigenes. In general, 1,015 (1,015/1,452); 2,127 (2,127/4,157); 1,958 (1,958/3,795); and 132 (132/232) unigenes from subgroup a–d were assigned to 187, 207, 210, and 92 KEGG pathways, respectively (Table S10). This detailed functional classification is valuable in further investigating their roles in parasite-host interactions.

## Discussion

The aim of the present study was to markedly enlarge the current dataset of *D. immitis* transcripts and identify some groups of genes predicted to play key roles in essential biological processes in the parasite. The integrated genomic-bioinformatic approach produced 20,810 assembled unigenes (longer than 300 bp). We found approximately 10 times more heartworm unigenes than the EST clusters generated by a previous study [21], which constitutes a significant contribution to current databases. A total of 15,698 CDSs were inferred from the present transcriptome dataset. Importantly, about 85% of the unigenes had orthologs/homologs in public databases, which means a much higher percentage of annotations compared to those (less than 50%) in similar transcriptome studies of other animal-parasitic helminths [27], [28], [54], [55]. The high level annotations are due to the fact that all eight public databases were selected for acquiring complete functional information, based on two levels of sequence similarity, namely sequence-based and domain-based alignments. Another reason for this result is the very high mean length of assembled unigenes, resulting from the full-length transcriptome assembly approach through Trinity [36].

Only 0.04% clean reads were mapped to *Wolbachia* genome, and only 12 reads were mapped to *Wolbachia* genes (summarized in Table S2). In addition, 34 unigenes from our dataset were considered as host-associated transcripts. Given that the lateral gene transfer (LGT) may occur from *Wolbachia* endosymbionts to heartworms, or from dogs to heartworms, further experiments are needed to investigate whether those *Wolbachia*-mapped sequences and the putative ‘dog-specific’ unigenes represent LGT events.

Six-hundred proteins inferred from *D. immitis*, which associate with 14 immune-system pathways may be of importance in host immune responses or the modulation thereof (Table S5). Of particular note are the proteins involved in the complement cascade as a part of the pathway namely ‘Complement and coagulation cascades, ko04610’. The complement system is an essential component of innate immunity, as well as an important modulator of adaptive immune responses [56], [57]. Previous works demonstrated the effects of complement proteins (e.g., C3 and C5) on microfilariae of *D. immitis*, which mediate the innate immunity by facilitating adherence between the worm cuticular surface and granulocytes (especially eosinophils) [58], [59]. Hamada (1988) suggested that IgG antibody binding to *D. immitis* microfilariae increased in the presence of purified C1q (a subcomponent of the first component of complement) [60]. In another study, deposits of complement and IgG, presumably associated with complex formation, were observed in some heartworm-infected dogs [61]. However, the interaction between adult heartworms and complement components of hosts is still unclear. Although the significance or role of the unigenes of adult heartworms involved in the complement system remains to be established, they may associate with modulation and modification of the responses of lymphocytes to stimuli [56].

An initial trial with the crude detergent extract of heartworm intestine showed that *D. immitis* gut-associated antigens were not recognized by normally infected hosts, but induced partial protection against a challenging infection [53]. However, there are currently no reports investigating the intestinal gene repertoire of *D. immitis* on a molecular level. Based on the *D. immitis* transcriptome data, the present study identified that 1,101 unigenes had similarities to intestine-expressed genes from at least one of the three queried nematodes, and the distribution of the matches partly reflects the phylogenetic relationships among them. The most matches were shared by *A. suum* and *D. immitis*. The possible reason may be that both *A. suum* and *D. immitis* are clade III nematodes. *H. contortus* and *C. elegans*, on the other hand, are classified into clade V, with an evolutionary distance from clade III *D. immitis* estimated to be ∼350 million years [62]. Surprisingly, the distribution of the matches failed to reflect the feeding pattern differences among those nematodes. Both *D. immitis* and *H. contortus* derive their nutrition from host blood or serum, and most likely share some metabolic processes involved in digestive and assimilative functions. However, both the intestinal nematode *A. suum*, which presumably feeds on semi-digested contents in the host gut [35], and the free-living bacterivorous *C. elegans* shared more gut-expressed gene homologs with the blood-feeding *D. immitis*. This may be due to the relatively small intestinal transcriptome of *H. contortus* and the evolutionary distance between *H. contortus* and *D. immitis*. Indeed, intestinally-expressed genes fall into two broad classes, including widely expressed ‘housekeeping’ genes and genes that are either intestine-specific or significantly intestine-enriched [46]. The 60 unigenes, which may be involved in the core cellular and physiological intestinal functions common to the four nematode species, hypothetically associate with the former class. On the other hand, the unigenes that only mapped to single intestine transcriptome are likely associated with the latter class, implying that the ‘hidden antigens’ should be expected to be good vaccine candidates for *D. immitis*. Further gene expression profiling and experimental validation will be needed to test our hypothesis and investigate the intestinal-enriched genes of *D. immitis*.

The presence of the endosymbiont *Wolbachia* sp. has been observed in the great majority of filarial nematode species, including *D. immitis* and *B. malayi*, while it appears to be absent in some filarial species and non-filarial worms [21], [63], [64]. There is convincing evidence that in those filarial species that harbor *Wolbachia*, they are essential for long-term survival and reproduction of filarial species [65], [66]. Investigation of the molecular basis and important pathways and proteins involved in the filaria-*Wolbachia* relationship is required for the development of drugs to disrupt this symbiosis [67]. To elucidate these symbiotic mechanisms, previous work has focused on the comparative genomic analyses between filarial hosts and the endosymbionts [67], [68]. Another experimental approach is to discover genes that are differentially expressed in the filarial host after depletion of their *Wolbachia* endosymbionts [67], [69]. In the present study, combining our *D. immitis* transcriptome data with the Nembase4 dataset, we employed comparative approaches to mine genes of importance in the heartworm-*Wolbachia* symbiosis. *Wolbachia* is not necessary for the survival of all filarial species, and endosymbiont-free species may synthesize the essential compounds themselves or take them up from the environment rather than depending on *Wolbachia* (reviewed in [67], [70]), suggesting that gene functions and expression of *Wolbachia*-containing filarial species may in some aspects differ significantly from their *Wolbachia*-free relatives. These differences may reflect either the phylogenetic distance between them, or the adaptation of symbiotic relations. We compared our *D. immitis* transcriptome data with the EST repertoires of other *Wolbachia*-containing filarial species, non-*Wolbachia* filarial species, and non-filarial nematodes, respectively. Thus, we revealed that 3,795 (18.2% of total *D. immitis* unigenes) potentially specific unigenes are only shared by filarial species that harbor *Wolbachia*, and these unigenes may be associates with the adaptation of symbiotic mechanisms. Of the 3,795 unigenes, 1,958 acquired KO annotations and were assigned to 210 pathways. Filarial worms live in a broad set of host species or different host tissues, and it may be difficult to ascribe a single, exclusive function to *Wolbachia* [65]; therefore, it is possible that the effects of *Wolbachia* may vary between different *Wolbachia*-containing species [65], and affect gene expression of many distinct pathways [67]. Due to the relatively small gene repertoires of *Wolbachia*-free filarial species, their ‘filtering effect’ for our unigenes was not strong enough, which means that some non-specific genes for *Wolbachia*-containing filarial were also included in the 3,795 unigenes. Further comparison between the discovered potential symbiosis-specific unigenes and more transcriptomic data sets of filarial species will be needed to discover the drug-targetable genes of *D. immitis* that indicate biochemical or physiological dependencies in the *Wolbachia*-filaria relationship.

In conclusion, the present study presents a large-scale characterization of the adult *D. immitis* transcriptome and significantly enlarges the currently known gene repertoire of *D. immitis*. This work will also assist in efforts to decode the genome of *D. immitis* [20], [71]. We have also shown that the comparative analysis of the heartworm transcriptome data with those of other nematode species can yield interesting information not evident in analyses of single species.

## Supporting Information

Figure S1 **Comparison of unigene lengths between hit and no-hit unigenes by searching against the Nr database.**
(TIF)

Figure S2 **Gene ontology (GO) classification of putative intestinal-expressed peptides inferred from** ***D. immitis*** **transcriptome.**
(TIF)

Figure S3 **Gene ontology (GO) classification of putative peptides inferred from four subgroups.** (a) Subgroup a. (b) Subgroup b. (c) Subgroup c. (d) Subgroup d.
(TIF)

Table S1 **Four groups of EST-clusters from Nemabase4 data.**
(XLS)

Table S2 **Summary of** ***Dirofilaria immitis*** **clean reads mapped to** ***Wolbachia*** **genome/genes.**
(XLS)

Table S3 **Assessment of assembly.**
(XLS)

Table S4 **Annotations against Pfam.**
(XLS)

Table S5 **Summary of KEGG pathways information for adult** ***Dirofilaria immitis*** **transcriptome.**
(XLS)

Table S6 **Unigenes associated with ‘Cardiovascular Diseases’ KEGG pathway.**
(XLS)

Table S7 **Putative intestinal-expressed unigenes of** ***Dirofilaria immitis*** **.**
(XLS)

Table S8 **KEGG pathways for 977 putative intestinal-expressed peptides of** ***Dirorilaria immitis*** **.**
(XLS)

Table S9 **Unigenes in subgroups a–d.**
(XLS)

Table S10 **a. KEGG pathways for 1,015 putative peptides from subgroup a.**
(XLS)

File S1 **The 34 unigenes which has BLAST matches with the host (canine) genes but nematode genes in NCBI.**
(XLS)
