# Supplementary figures and images for: Novel Insights into the Transcriptome of Dirofilaria immitis
Source: PLoS One. 2012 Jul 23;7(7):e41639. doi: 10.1371/journal.pone.0041639 (PMC3402454; doi:10.1371/journal.pone.0041639)

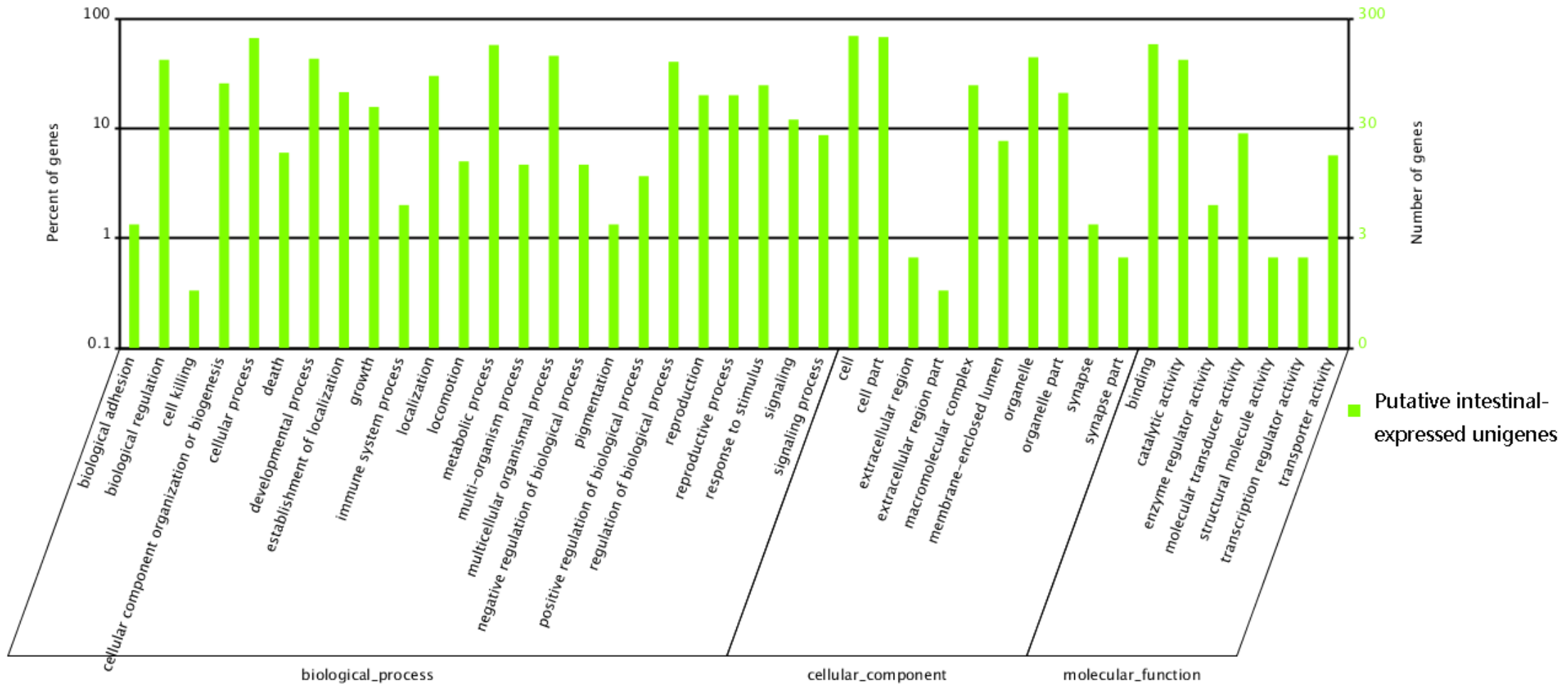

Supplement: Figure S2 — Gene ontology (GO) classification of putative intestinal-expressed peptides inferred from D. immitis transcriptome. (TIF) [file pone.0041639.s002.tif]

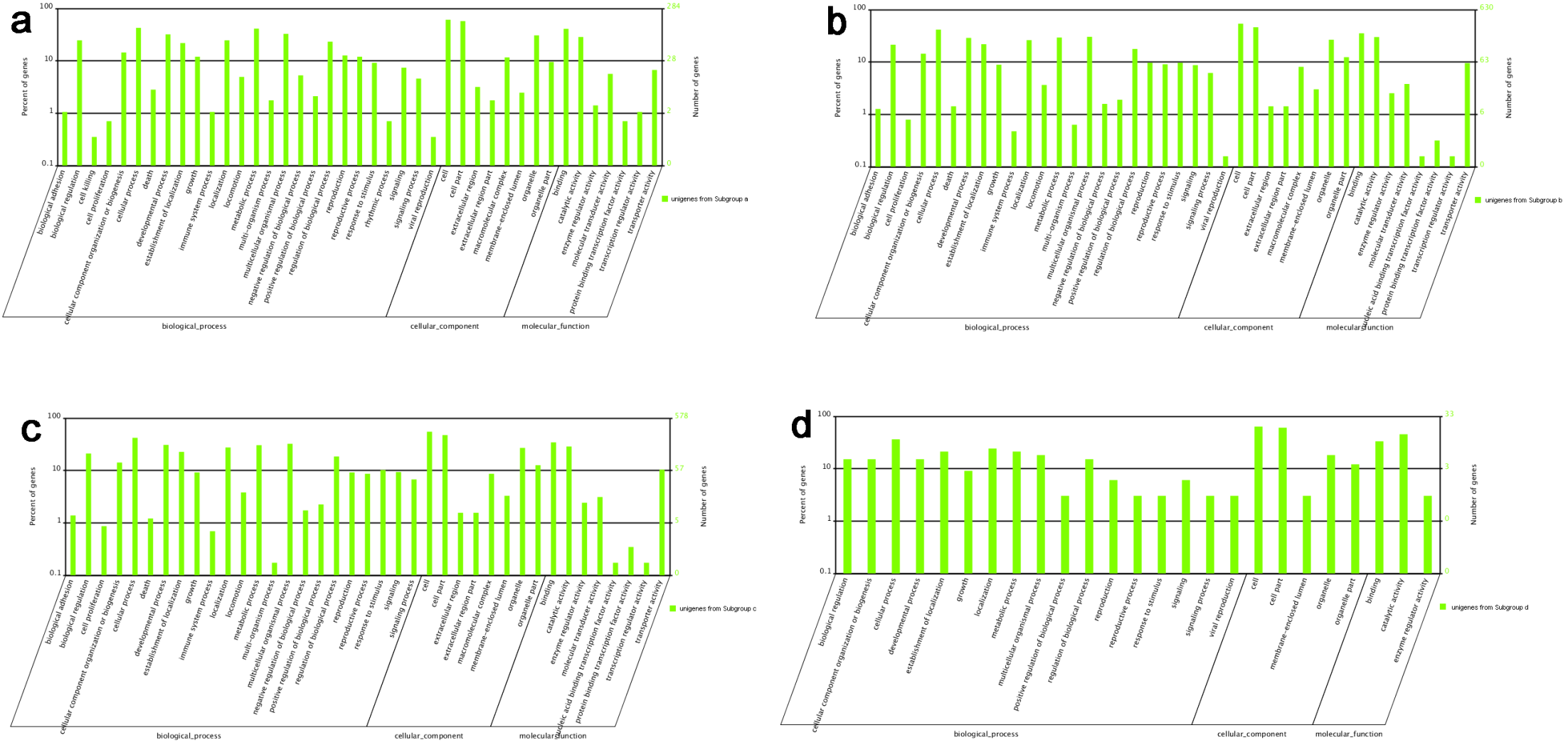

Supplement: Figure S3 — Gene ontology (GO) classification of putative peptides inferred from four subgroups. (a) Subgroup a. (b) Subgroup b. (c) Subgroup c. (d) Subgroup d. (TIF) [file pone.0041639.s003.tif]
